# Early Transcriptomic Response to Phosphate Deprivation in Soybean Leaves as Revealed by RNA-Sequencing

**DOI:** 10.3390/ijms19072145

**Published:** 2018-07-23

**Authors:** Houqing Zeng, Xiajun Zhang, Xin Zhang, Erxu Pi, Liang Xiao, Yiyong Zhu

**Affiliations:** 1 College of Life and Environmental Sciences, Hangzhou Normal University, Hangzhou 310036, China; zhangxiajun1992@163.com (X.Z.); z123767371@126.com (X.Z.); pierzaixian001@aliyun.com (E.P.); 2 College of Resources and Environmental Sciences, Nanjing Agricultural University, Nanjing 210095, China; 2016203037@njau.edu.cn

**Keywords:** soybean (*Glycine max*), phosphate deprivation, leaf, RNA-sequencing, hormone, calcium signaling, transcriptional response, kinase, metabolism, cis-element

## Abstract

Low phosphate (Pi) availability is an important limiting factor affecting soybean production. However, the underlying molecular mechanisms responsible for low Pi stress response and tolerance remain largely unknown, especially for the early signaling events under low Pi stress. Here, a genome-wide transcriptomic analysis in soybean leaves treated with a short-term Pi-deprivation (24 h) was performed through high-throughput RNA sequencing (RNA-seq) technology. A total of 533 loci were found to be differentially expressed in response to Pi deprivation, including 36 mis-annotated loci and 32 novel loci. Among the differentially expressed genes (DEGs), 303 were induced and 230 were repressed by Pi deprivation. To validate the reliability of the RNA-seq data, 18 DEGs were randomly selected and analyzed by quantitative RT-PCR (reverse transcription polymerase chain reaction), which exhibited similar fold changes with RNA-seq. Enrichment analyses showed that 29 GO (Gene Ontology) terms and 8 KEGG (Kyoto Encyclopedia of Genes and Genomes) pathways were significantly enriched in the up-regulated DEGs and 25 GO terms and 16 KEGG pathways were significantly enriched in the down-regulated DEGs. Some DEGs potentially involved in Pi sensing and signaling were up-regulated by short-term Pi deprivation, including five SPX-containing genes. Some DEGs possibly associated with water and nutrient uptake, hormonal and calcium signaling, protein phosphorylation and dephosphorylation and cell wall modification were affected at the early stage of Pi deprivation. The cis-elements of PHO (phosphatase) element, PHO-like element and P responsive element were present more frequently in promoter regions of up-regulated DEGs compared to that of randomly-selected genes in the soybean genome. Our transcriptomic data showed an intricate network containing transporters, transcription factors, kinases and phosphatases, hormone and calcium signaling components is involved in plant responses to early Pi deprivation.

## 1. Introduction

As a non-substitutable macronutrient, phosphorus (P) is essential for plant growth and development by being part of fundamental bio-molecules and participating in various cellular activities. However, plants frequently suffer from P deficiency, which is a limiting factor for crop productivity worldwide, because of the low availability of the available form of P (phosphate, Pi) in soils, especially in acid soils [1,2]. Substantial use of Pi fertilizers is the main solution for this problem but the reserves of Pi rock (the primary source of Pi fertilizers) are finite and the cost of Pi fertilizers is high. In order to improve Pi acquisition and use efficiency of crop plants, many studies have been conducted to understand the physiological, biochemical and molecular mechanisms of plant adaptation and tolerance to low Pi stress [3,4,5,6].

Plants have a series of adaptive strategies to cope with low Pi stress, such as changes in shoot-to-root biomass ratio, exudation of organic acids, secretion of acid phosphatases, induction of high-affinity Pi transporters (PHTs), association with soil microorganisms, release of Pi from vacuolar stores and the optimization of internal Pi utilization by metabolic changes [7,8,9]. A large number of Pi-responsive genes have been functionally identified to coordinate these Pi deficiency responses. For example, in Arabidopsis, the MYB-CC (MYB-coiled–coil) transcription factor *PHR1* and its close homologs *PHR1-LIKE 1* (*PHL1*) and *PHL2* are considered to be the central signaling components controlling transcriptional and metabolic responses to variations in Pi supply [10,11,12]; SPX-MFS (SPX-Major Facilitator Superfamily) protein VPT1 (Vacuolar Phosphate Transporter 1, also named PHT5;1) is regarded to mediate vacuolar Pi sequestration, which is critical for plant acclimation to varying Pi availability in the environment [13,14,15]; microRNAs are important players in controlling Pi transport by regulating the expression levels of some key components in Pi signaling networks, such as *PHO2* and *NLA* (*Nitrogen Limitation Adaptation*) [16,17,18].

Transcriptomic analyses by microarray and recently developed high-throughput sequencing methods provide a useful tool for holistic understanding of the transcriptional responses under Pi deficiency in the post-genome era. Significant progress has been made on model plant Arabidopsis [19,20,21,22], rice [23,24] and some other plants [25,26,27]. Numerous genes were shown to be responsive to Pi deprivation, including genes involved in Pi acquisition and recycling, hormonal signaling and transcriptional regulation. These findings also support previous physiological and biochemical studies [28,29]. However, most of these studies are based on mid-term or long-term Pi deprivation. Early transcriptional responses to Pi deprivation are still largely ambiguous.

Soybean is an important leguminous crop for providing cooking oil and protein-rich food for humans. Low Pi availability is considered to be the most limiting nutrient condition for its production [30]. Some studies have been conducted to demonstrate the mechanisms of Pi deficiency response and tolerance. For instance, *GmACP1* and *GmACP2* both encoding an acid phosphatase was found to contribute to low Pi tolerance by combining quantitative trait loci (QTL) linkage, comparative transcriptome and genome-wide association analyses [31,32]; *GmPHT1;4* (also named *GmPT5*) is critical for maintaining Pi supply in nodules by transporting Pi from roots to nodules [33]; three metabolism pathways including methane metabolism, phenylalanine metabolism and phenylpropanoid biosynthesis were found to be enriched in the common Pi-responsive genes between a low-Pi tolerant soybean accession and a low-Pi sensitive soybean accession [34]. Recently, we studied the transcriptomic responses to medium-term Pi deficiency (7 days) by digital gene expression deep sequencing and identified a total of 1612 differentially expressed genes (DEGs) in soybean roots [35]. However, the early signaling events in low Pi stress responses remain elusive. Leaves are important sources of the shoot-to-root systemic signals in Pi deficiency response, such as phloem mobile RNAs, proteins, sugars, Pi and other metabolites [4,9] but the sensing and signaling of Pi deficiency in leaves is largely unclear. In order to capture early transcriptional response to Pi deprivation in soybean leaves, we measured the transcriptional response to short-term (24 h) Pi deprivation by RNA-Seq. The DEGs and signaling components identified here represent new candidates for understanding the molecular mechanisms of early Pi stress responses in leaves and the improvement of Pi stress tolerance in soybean.

## 2. Results

### 2.1. Transcriptome Profiling by RNA-Seq of Soybean Leaves in Response to Short-Term Pi Deprivation

**Figure 1:**
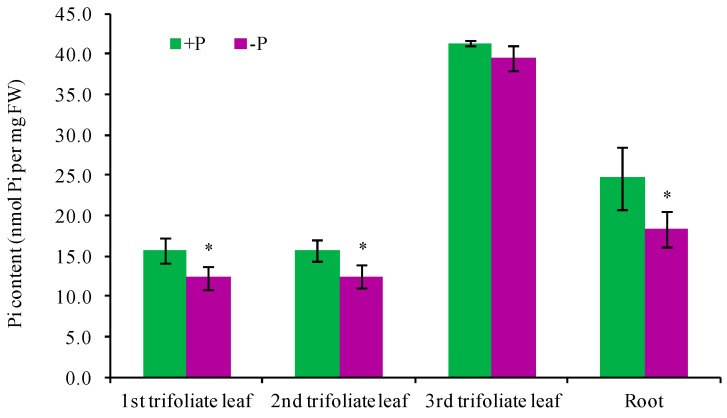
Pi concentration in the first trifoliate leaf (1st TL), the second TL (2nd TL), the third TL (3rd TL) and the roots of soybean seedlings after being subjected to Pi-deficiency treatment for 24 h. Data presented are mean ± SD (standard deviation) of three independent experiments. Asterisk indicates the statistically significant difference between control (+P) and Pi-deficiency (−P) treatments (Student’s *t*-test, * *p* < 0.05) (Microsoft Excel 2007).

To assess the global transcriptome profile of soybean leaves in response to short-term Pi deprivation, we performed RNA-seq analyses following Pi deprivation for 24 h, a time point where the Pi concentration of Pi-deprived tissues decreases [23,36]. After Pi deprivation treatment for 24 h, Pi concentration in the roots, the first and the second trifoliate leaves of soybean significantly decreased but there was no significant difference for the third trifoliate leaves (Figure 1). Two biological replicates of RNA-seq were included for both Pi-sufficient leaves (PSL) and Pi-deprived leaves (PDL), and, therefore, a total of four libraries were constructed. By Illumina’s deep sequencing, a total of 38.8 to 49.6 million reliable clean reads were obtained from each library after excluding the low-quality reads (Table S1). Most of the clean reads (69.5–74.1%) from each library uniquely mapped to the soybean reference genome (v2.0). The correlation of the two biological replicates for PDL and PSL was calculated to determine the variability between the replicates. The Pearson’s correlation (R value) of the two comparisons was both around 85% (Figure S1), indicating a high correlation between the biological replicates. The abundance of transcripts from each gene mapped was measured in terms of the fragments per kilobase of transcript per million mapped reads (FPKM). A total of 39,505 gene loci were detected in PDL and/or PSL (Table S2).

Differential expression analysis showed that a total of 533 loci were differentially expressed, including 36 putative mis-annotated loci and 32 putative novel loci (Tables S3–S5). Here, mis-annotated loci were considered as loci assembled by Cufflinks but spanning two or more loci annotated in the soybean genome assembly. This is often due to the incorrect annotation of the initial gene model or annotated genes being too close together to be resolved. Unannotated loci (i.e., the novel loci) are an assembled group of reads not overlapping with any known loci annotated in the soybean genome assembly. Among the DEGs that have been annotated previously, 270 were up-regulated and 195 were down-regulated by short-term Pi deprivation (Figure S2). Interestingly, transcripts from nine DEGs were found only in PDL, while transcripts from 12 DEGs were found only in PSL (Table 1). The exclusive expression patterns suggest that these genes could have a potential role in plant response to short-term Pi deprivation.

### 2.2. Verification of RNA-Seq Data by qRT-PCR

**Figure 2:**
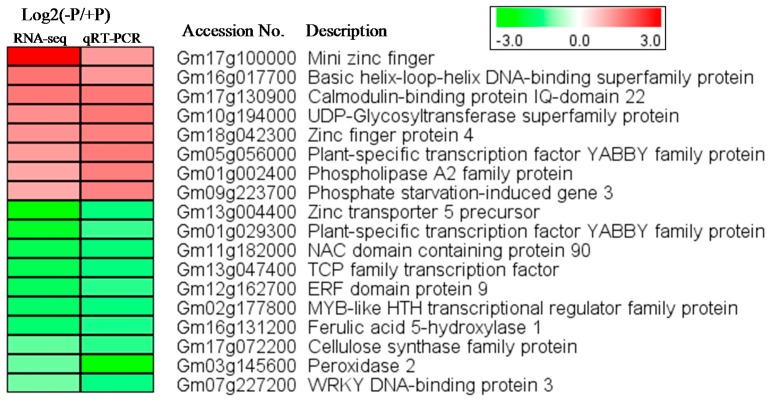
Heatmap of the expression of 18 randomly selected differentially expressed genes (DEGs) as revealed by RNA-seq and qRT-PCR. The intensities of the color represent the fold changes in log2 values according to RNA-seq data or qRT-PCR results. Red color indicates induction and green color indicates repression. Accession number “Glyma.” is named as “Gm” for short. −P refers to PDL and +P refers to PSL.

To validate the reliability of the RNA-seq data, 18 DEGs were randomly selected and investigated by qRT-PCR. The fold changes of these genes under short-term Pi deficiency observed by qRT-PCR were similar to that revealed by RNA-seq (Figure 2), indicating that the RNA-seq data obtained in this study is reliable. However, there were differences between RNA-seq and qRT-PCR results for some genes. For example, the induction of *Gm17g100000* transcripts appeared much stronger when detected by RNA-seq than by qRT-PCR, while the repression of *Gm03145600* transcripts appeared much stronger when detected by qRT-PCR than by RNAseq. These discrepancies may be due to the facts that the samples used for RNA-seq and qRT-PCR were not the same and there would be inevitable differences between different batches of samples and perhaps some primer pairs used in qRT-PCR were not optimal for detecting the target transcripts.

### 2.3. Enrichment Analysis of Gene Ontology (GO) Functional Annotation and KEGG Pathways of Differentially Expressed Genes (DEGs)

Half of the DEGs (266/533) can be assigned to 183 GO terms (Table S3). GO enrichment analysis revealed that 29 and 25 GO terms were significantly enriched in the up-regulated DEGs and the down-regulated DEGs, respectively (Tables S6 and S7). For the up-regulated DEGs, carbohydrate metabolic process, cellular glucan metabolic process and lipid metabolic process were the three most significantly enriched GO terms within biological processes; each term contains at least three DEGs (Table S8). For the down-regulated DEGs, dTMP (deoxy-thymidine monophosphate) biosynthetic process, glycine biosynthetic process and nucleotide biosynthetic process were the three most significantly enriched GO terms within biological processes; each term contains at least two DEGs (Table S9). In addition, 15.6% of the DEGs (83/533) can be assigned to 152 KEGG pathways (Table S3). Eight and 16 KEGG pathways were significantly enriched in the up-regulated DEGs and the down-regulated DEGs, respectively (Table S10). Ether lipid metabolism, cutin, suberine and wax biosynthesis and pentose and glucuronate interconversions were the three most significantly enriched KEGG pathways in the up-regulated DEGs; each contains two to three DEGs. Drug metabolism-cytochrome P450, chemical carcinogenesis and metabolism of xenobiotics by cytochrome P450 were the three most significantly enriched KEGG pathways in the down-regulated DEGs; each contains three to four DEGs.

### 2.4. Genes Potentially Involved in Pi Signaling and Utilization Are Induced by Short-Term Pi Deprivation in Soybean Leaves

In this study, at least 13 classical Pi-responsive genes that are potentially involved in Pi signaling and utilization were found to have changed expression in response to short-term Pi deprivation (Table 2). These genes included five genes encoding SPX domain-containing proteins, two purple acid phosphatase (*PAP*) genes, three phospholipase genes, one glycerol-3-phosphate permease (*G3Pp*) gene, one sulfolipid sulfoquinovosyldiacylglycerol (*SQD*) gene and one gene encoding subfamily IIIB acid phosphatase (*Gm16g220900*). All of these genes were significantly up-regulated by Pi deprivation, with the exception that *Gm16g220900*—which potentially encodes a class IIIB acid phosphatase—was repressed.

**Figure 3:**
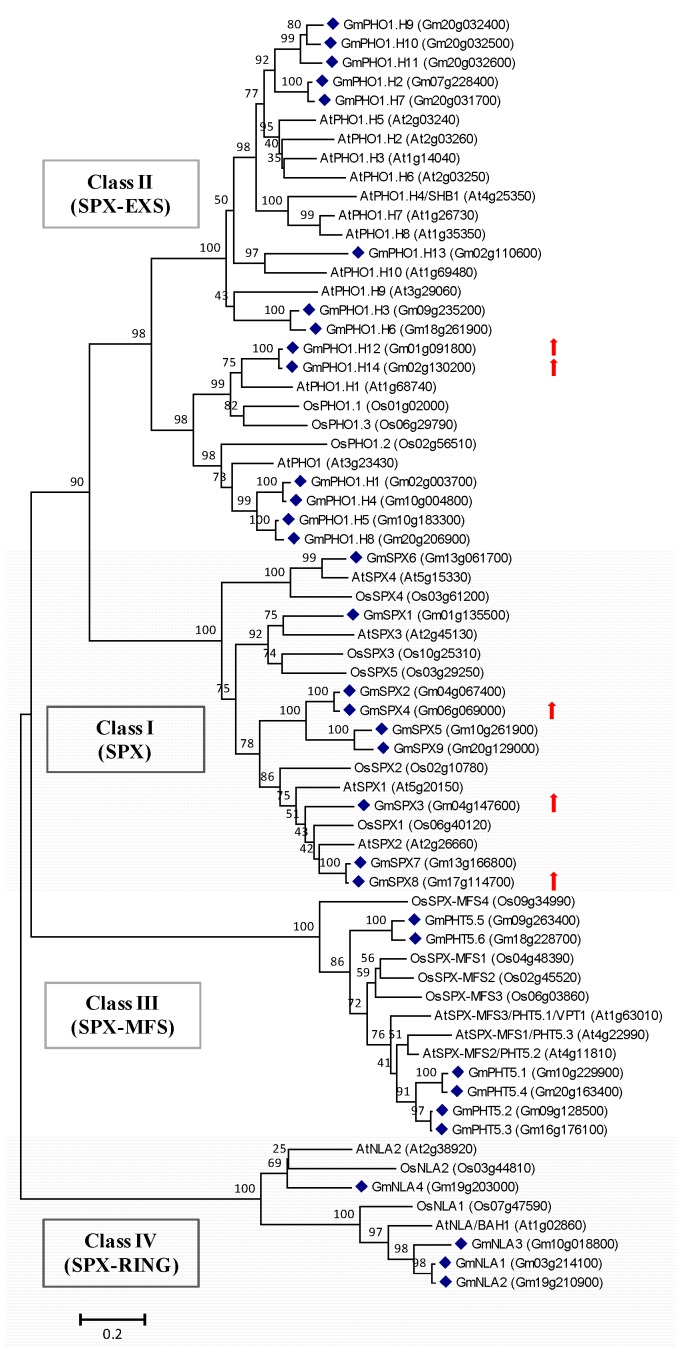
Unrooted phylogenetic tree of the SPX domain-containing proteins in soybean (*Glycine max*), Arabidopsis (*Arabidopsis thaliana*) and rice (*Oryza sativa*). The alignment for the phylogenetic tree was performed with ClustalW using full-length amino acid sequences. The phylogenetic tree was created with the MEGA6 software and the neighbor-joining method with 1000 bootstrap replications. The bar indicates the relative divergence of the sequences examined. The red arrow indicates the up-regulation of *SPX* genes upon short-term Pi deprivation by RNA-seq in the present study. Soybean SPX domain-containing proteins are marked with blue diamond.

SPX-containing proteins can be classified into four sub-families based on the presence of additional domains in their structure, namely the SPX, SPX-EXS, SPX-MFS and SPX-RING families. They are conserved in higher plants and are essential for Pi signaling and utilization [4,37,38]. Previously, nine SPX proteins and 14 SPX-EXS proteins have been identified in the soybean genome [39,40]. Here, an additional six *SPX-MFS* genes and four *SPX-RING* genes were identified in the soybean genome based on homology searches using Arabidopsis counterparts (AtPHT5;1 and AtNLA1) (Tables S11 and S12). Alignments with the sequences of SPX-MFS proteins and SPX-RING proteins were performed by ClustalW (Figures S3 and S4), suggesting that the SPX, MFS and RING domains are conserved among these proteins. Phylogenetic analysis indicated that soybean SPX-containing proteins are closely related to their homologs in Arabidopsis and rice (Figure 3), suggesting their conserved functions in land plants.

### 2.5. Expression of Genes Potentially Involved in Transportation of Water, Sugars and Mineral Nutrients Is Altered by Short-Term Pi Deprivation

Twenty-seven transporter genes were found to be differentially expressed in Pi-deprived soybean leaves (Table 3). Among them, several genes potentially involved in transportation of water; sugar; sulfate; and copper were up-regulated, while three genes potentially involved in zinc/iron transport were down-regulated by short-term Pi deprivation. In addition, one nitrate transporter gene (*Gm18g127200*) and one malate transporter gene (*Gm11g179100*) were down-regulated by Pi deprivation. The responsiveness of these transporter genes suggested that transport and allocation of water, sugars and nutrients are quickly altered by Pi deprivation in order to adapt to the stressed condition.

### 2.6. Genes Linked to Ca^2+^ and Hormonal Signaling are Regulated by Short-Term Pi Stress

At least 10 genes differentially expressed in Pi-deprived soybean leaves were putatively linked to Ca^2+^ signaling (Table 4). In addition, at least 15 DEGs are potentially involved in the transport and signaling of diverse hormones, including auxin, cytokinin, gibberellin (GA), brassinosteroids (BRs), jasmonate and ethylene (Table 4). Among these genes, DEGs related to GA, BR and jasmonate signaling were all up-regulated by Pi deficiency. The expressional changes of these hormone-related genes suggested that short-term Pi deficiency could affect hormone synthesis, transport and sensitivity.

### 2.7. Diverse Transcription Factor Family Genes Are Responsive to Short-Term Pi Deprivation in Soybean Leaves

Here, expression of at least 31 transcription factor genes was affected by short-term Pi deprivation in soybean leaves; among them, 14 were up-regulated and 17 were down-regulated (Table 5). Several transcription factors are also potentially involved in hormonal signaling. For example, *Gm14g127400* encoding a BES1/BZR1 homolog protein is potentially involved in BR signaling and *Gm05g144500* encoding an RR protein is potentially involved in cytokinin signaling (Table 4). These differentially expressed transcription factors belong to diverse families, such as MYB, WRKY, NAC (NAM, ATAF, and CUC), ERF (ethylene response factor), bHLH (basic helix-loop-helix), TCP, bZIP (basic leucine zipper domain), HD-ZIP (homeodomain leucine zipper), YABBY and zinc finger proteins (ZFPs) of various types (C2H2, C3H, B-box, DOF and HD). MYB/MYB-like, bHLH, C2H2 ZFP and YABBY were the most abundant transcription factor families differentially expressed; each of them contained at least three DEGs. Interestingly, four DEGs encoding bHLH transcription factors were all up-regulated, whereas two genes encoding TCP transcription factors were all repressed by Pi deprivation.

### 2.8. Short-Term Pi Deprivation Modifies the Expression of Genes Encoding Diverse Protein Kinases and Phosphatases

**Figure 4:**
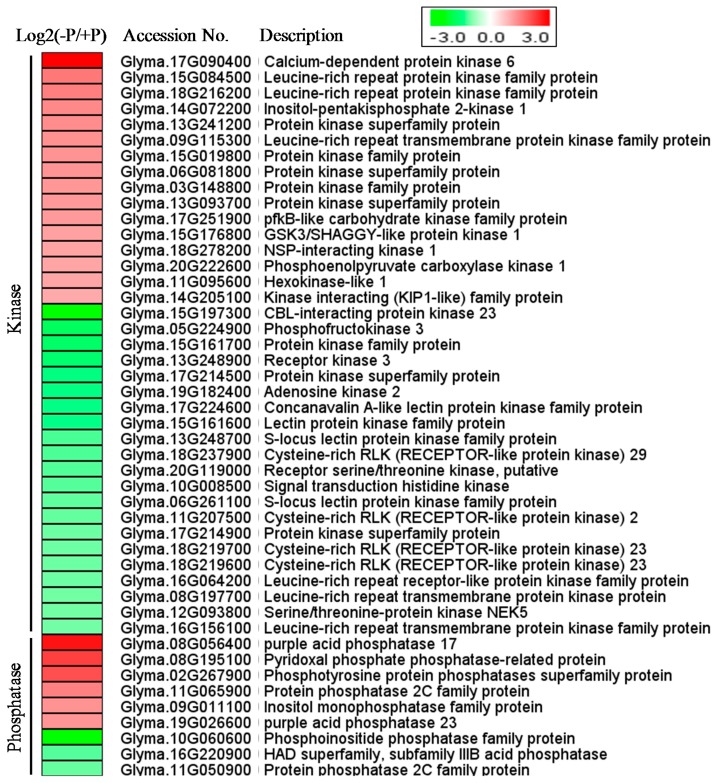
Heatmap of DEGs that are putative protein kinases and phosphatases. The intensities of the color represent the fold changes in log2 values according to RNA-seq data. Red color indicates induction and green color indicates repression. Accession number “Glyma.” is named as “Gm” for short. −P refers to PDL and +P refers to PSL.

In this study, at least 31 protein kinase genes and nine phosphatase genes (including three acid phosphatase genes listed in Table 2) were found to be responsive to short-term Pi deprivation (Figure 4). These DEGs potentially encode protein kinases of diverse families. In addition, most of the DEGs encoding potential phosphatases were up-regulated by Pi deprivation. However, a gene encoding phosphoinositide phosphatase (*Gm10g060600*) and a PP2C gene (*Gm11g050900*) were repressed by Pi deprivation, suggesting their differential roles in Pi-deprivation response.

### 2.9. The Expression of Genes Associated with Metabolism Is Affected by Short-Term Pi Deprivation in Soybean Leaves

**Figure 5:**
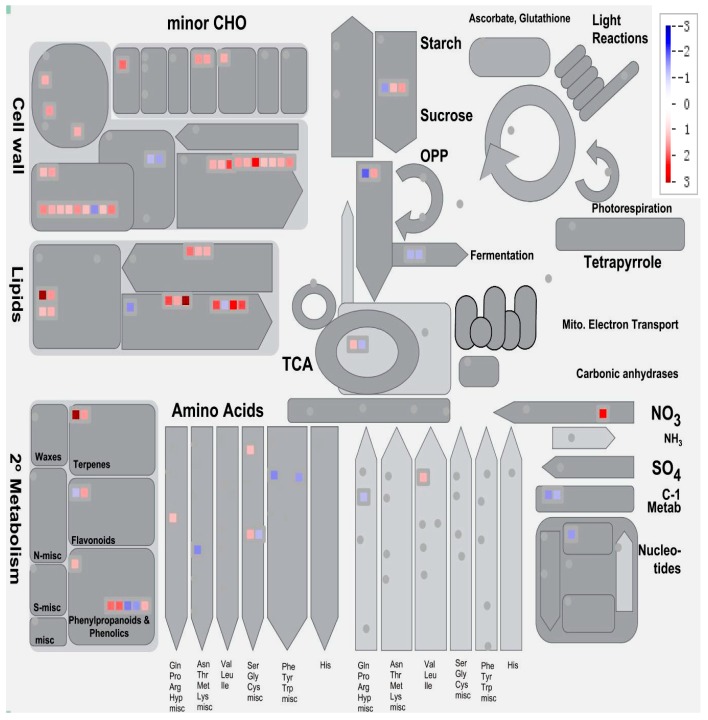
Mapman representation of DEGs involved in metabolism in soybean leaves upon short-term Pi deficiency. The results are the mean of two biological replicates. All results are shown on a log2 scale for corresponding FPKM ratios. Red color represents up-regulated genes and blue color represents down-regulated genes. TCA: tricarboxylic acid; OPP: oxidative pentose phosphate.

At least 77 DEGs were found to be associated with metabolism and the majority of them (72.7%) were up-regulated by short-term Pi deprivation (Figure 5, Table S13). Of the 15 DEGs potentially involved in lipid metabolism (including glycolipid synthesis, sulfolipid synthesis, fatty acid synthesis and elongation and lipid degradation), most were up-regulated under Pi deprivation. Ten DEGs potentially involved in cell wall degradation (including 1,4-β-mannan endohydrolases, pectate lyases and polygalacturonases) were all up-regulated by Pi deprivation. Seven of the 10 DEGs that are potentially involved in secondary metabolism of metabolites like carotenoids, terpenoids, isoflavonols and phenylpropanoids, were up-regulated by Pi deprivation. Moreover, nine DEGs were found to be potentially involved in the synthesis or degradation of some amino acids, like serine, cysteine, arginine, lysine, phenylalanine, tryptophan and proline (Table S13). Four of them were up-regulated and five were down-regulated by Pi deprivation, suggesting a complex regulation of amino acid metabolism under Pi deficiency and the different roles of these amino acids in Pi deficiency acclimations. In addition, nearly all of the 10 DEGs potentially involved in cell wall modification, such as expansins, xyloglucan endotransglycosylases, endoxyloglucan transferases and pectinesterases were up-regulated by short-term Pi deprivation. However, two DEGs potentially involved in cell wall cellulose synthesis (encoding cellulose synthases) were down-regulated by short-term Pi deprivation. These results suggest that plant metabolic acclimation to Pi deprivation stress occurs rapidly after encountering the stress.

### 2.10. Identification of Pi-Responsive Cis-Regulatory Elements in the Promoters of DEGs

**Figure 6:**
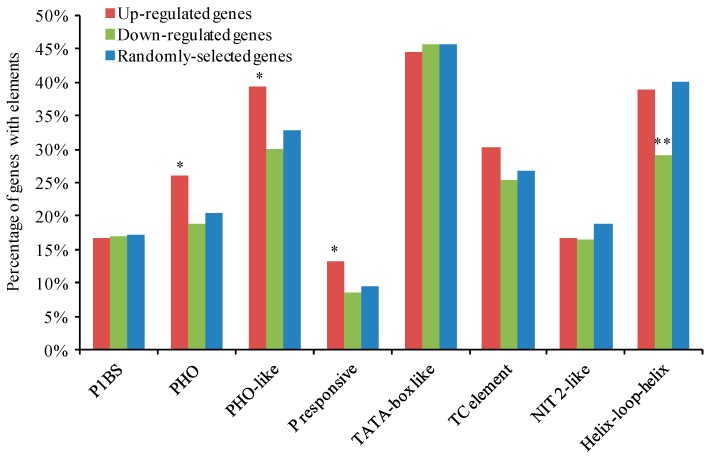
The occurrence of Pi-related cis-elements previously identified as common to Pi-responsive genes in the promoter regions (1000 bp) of up-regulated DEGs, down-regulated DEGs and randomly-selected genes. Promoter regions of 250 genes randomly selected from all the chromosomes of soybean genome were used as control. 288 and 213 promoter regions were acquired for up-regulated and down-regulated DEGs, respectively. The hypergeometric *p*-value was calculated online (http://systems.crump.ucla.edu/hypergeometric/index.php). Asterisk means significantly different from the genes with elements in the soybean genome that are predicted based on the results of randomly-selected genes (* *p* < 0.05, ** *p* < 0.01).

We examined the distribution of Pi-responsive cis-regulatory elements in the putative promoter regions up to 1000 bp upstream of the transcription start sites of DEGs. In total 501 promoter regions were obtained from 288 up-regulated loci and 213 down-regulated loci (Tables S14 and S15). Eight types of previously identified Pi-responsive cis-elements were found to exist in Pi-responsive genes [11,41,42,43]. The cis-elements of PHO (phosphatase) element, PHO-like element and P responsive element were present more frequently in the promoter regions of up-regulated DEGs as compared to that of randomly-selected genes in the soybean genome (Table S15, Figure 6). In addition, the Helix-loop-helix element was present less frequently in the promoters of down-regulated DEGs. However, no significant difference was found for P1BS, TATA-box like, TC element and NIT 2-like. These results suggest that the Pi-responsive cis-elements enriched in the promoters of DEGs may be involved in the transcriptional regulation events at the early stage of Pi deprivation stress.

## 3. Discussion

Because of the low availability of Pi in soils, understanding the molecular acclimation mechanisms under Pi stress is of critical importance for developing crops with enhanced P-use efficiency in modern agriculture. Several studies utilizing microarrays or deep sequencing have documented the genome-wide transcriptional responses of plants to Pi stress but most of these studies are based on mid-term or long-term Pi deficiency and the early responses remain poorly understood [9]. In this study, we measured the transcriptional responses of soybean leaves to short-term Pi deprivation using an RNA-Seq approach. A total of 533 loci were found to be responsive to early Pi deprivation. By comparison with the recent transcriptomic analyses in two recombinant inbred lines of soybean that have different Pi stress tolerance [32], 12 genes were found to be commonly responsive to Pi deprivation in leaves; five of them have similar responses to short-term or long-term Pi stress (Figure S5). In addition, a small portion of them (28/533) were found to be responsive to long-term Pi deprivation in soybean roots [35] (Figure S6). The majority of the 28 common genes showed a similar transcriptional response under short-term and long-term Pi deprivation in the different organs. There were 21 DEGs expressed exclusively in either PDL or PSL (Table 1) but none of them were found to be responsive to long-term Pi deprivation in soybean roots. This may suggest their different responses to short-term and long-term Pi deficiency in leaves and roots.

At least 13 genes which are commonly responsive to Pi deprivation in plants were affected by short-term Pi deprivation, including five *SPX* domain-containing genes, two PAP genes, three phospholipase genes, one *G3Pp* gene and one *SQD* gene (Table 2). Some of them, such as *PLDZ2*, *PAP13*, *PAP31*, *SPX3*, *SPX8* and *Gm09g223700* were also found to be responsive to long-term Pi deficiency [35,39,44,45]. Whether the other genes are responsive to long-term Pi deprivation or only responsive to early Pi deprivation needs further investigation. Replacement of phospholipids in internal cellular membranes with galactolipids and sulfolipids is an important adaptive mechanism under Pi deficiency, which have been found in many plant species [46,47]. Lipid remodeling is also considered to be related to mobilization of P from less-essential cellular components to be exported to growing sinks [8]. *PLDZ2* plays a critical role in galactolipid biosynthesis and thus facilitates Pi recycling from phospholipids [48]. SQD1 and SQD2 are Pi-responsive enzymes essential for sulfolipid biosynthesis under Pi-deficient conditions [49,50]; they also facilitate Pi recycling. In addition, PLDζ1 and PLDζ2 are Pi-responsive phospholipases that can hydrolyze phospholipids and thus contribute to Pi supply for Pi-deficient Arabidopsis [51]. Thus, the quick responses of these genes to Pi deprivation in leaves suggest that the replacement of phospholipids could be activated in a short time in order to adapt to Pi deficiency. *G3Pp* family genes which are potentially involved in transporting *G3P*, the hydrolysis products of diacylglycerol (a product of phospholipid breakdown), were found to be increased by Pi starvation in Arabidopsis [52]. Here, the homologs of these genes were also found to be induced by short-term Pi deprivation in soybean leaves but further researches are required to characterize their exact roles in Pi stress response and tolerance.

In this study, a total of 33 genes encoding SPX-containing proteins were found in soybean genome (Figure 3). The number is much higher than that in Arabidopsis (20) and rice (15) [38], which could be related to the whole-genome duplication events which occurred about 59 and 13 million years ago [53]. The early Pi deprivation-responsive *GmSPX3*, *GmSPX4* and *GmSPX8* are closely related to *SPX1* and *SPX2* in Arabidopsis and rice. In Arabidopsis and rice, *SPX1*, *SPX2*, *SPX4* and *SPX6* have been demonstrated to be involved in Pi sensing and signaling by inhibiting the transcriptional activity of PHR1 [54,55,56,57]. It has also been shown that overexpression of soybean *SPX1* and *SPX3* increases and decreases total P concentration in plant tissues, respectively, suggesting their contrasting roles in the regulation of Pi distribution in the plant [39,45]. Whether the short-term Pi deprivation-responsive *SPX* genes in soybean would function in Pi deficiency response by repressing the activity of conserved *PHR1* and/or by participating in other regulatory pathways deserve further investigation. In addition to *SPX* genes, two *SPX-EXS* genes *GmPHO1.H12* and *GmPHO1.H14* were also induced by short-term Pi deprivation. Their closely related homologs in Arabidopsis, such as *AtPHO1* and *AtPHO1.H1* are also responsive to Pi deficiency and are associated with Pi economy [37,58]. However, the functions of Pi-responsive SPX-containing proteins in soybean remain to be investigated in the future.

In addition to the genes that are potentially involved in Pi signaling and utilization, many genes possibly involved in transportation of water, sugars and other mineral nutrients were also altered by short-term Pi deprivation (Table 3). It has been known for a long time that Pi deficiency decreases leaf water potential and transpiration rate [59]. Thus, it would be interesting to determine whether the observed up-regulation of aquaporins could be a compensatory response for the lower hydraulic conductance under Pi deficiency. Enhancement of the uptake and translocation of sugars and sulfate could also promote the synthesis of galactolipids and sulfolipids to substitute for the decline of phospholipids under Pi deficiency [8]. Sugar is an important systemic signal for regulating Pi starvation responses and root system architecture [60]. In addition, the homeostasis of iron, zinc and copper could also be affected by Pi deficiency. The most abundant Cu proteins in green tissues are plastocyanin and Cu/Zn-superoxide dismutase (Cu/ZnSOD), which are associated with electron transfers in photosynthesis and the scavenging of stress-induced reactive oxygen species (ROS), respectively [61]. Therefore, the up-regulation of copper transporters may be a part of a mechanism to boost ROS scavenging and photosynthesis which are impaired by Pi deficiency. Both iron and zinc have been shown to accumulate in Pi-deficient leaves in Arabidopsis [21]. Thus, the down-regulated iron/zinc transporter genes shortly after Pi deficiency could impede the excessive accumulation of these metals in the long run.

In this study, at least 10 DEGs are potentially involved in Ca^2+^ signaling (Table 4). Ca^2+^ is a universal signal playing a critical role in plant responses to environmental stresses [62,63]. It is well-known that diverse external environmental stimuli can quickly trigger specific spatial-temporal patterns of changes in cytosolic Ca^2+^ concentration, which can be perceived and decoded by a series of Ca^2+^ sensors containing EF-hand motifs [64,65]. However, little information is available with respect to the effect of Pi deficiency on cytosolic Ca^2+^ levels. Considering the critical roles of the Ca^2+^ signal in plant responses to other nutrient stress, such as potassium and nitrate deficiencies [66,67], it is conceivable that Ca^2+^ signals might be an important player in Pi stress response. In addition, Ca^2+^ and Pi are incompatible ions, because Ca^2+^ can form insoluble compounds with phosphate derivatives at high levels. Changing the allocation patterns of Pi may also necessitate changes to the patterns for Ca^2+^. Thus, how Ca^2+^ signal is linked to Pi deficiency response should deserve further investigations.

Many hormones have been involved in Pi stress responses by regulating root development and architecture as well as shoot development [4]. Here 15 genes potentially involved in signaling of auxin, CK (cytokinin), GA, BRs, jasmonate and ethylene were responsive to short-term Pi deprivation (Table 4). It has been shown that bioactive GA levels are reduced by Pi deficiency, which leads to the accumulation of DELLA proteins and, therefore, modulates root system architecture and anthocyanin accumulation in leaves [68]. BRs are a class of plant polyhydroxysteroids playing a pivotal role in plant growth and development as well as a wide variety of environmental stress responses [69]. However, there is little information available on the role of BRs in Pi deficiency. Whether BRs are involved in regulating the Pi economy during leaf development under Pi stress remains to be examined. Initiation and expansion of soybean leaves were demonstrated to decline under Pi deficiency [70]. However, whether hormonal signals like auxin, BRs, CK are involved in these physiological processes remains to be answered. In addition, leaf senescence is usually accelerated under nutrient stress in order to enhance remobilization of nutrients from senescing leaves [8] but the exact roles of hormones like ethylene, CK, jasmonate, auxin in nutrient stress-induced leaf senescence remain to be investigated in the future.

Transcription factors are critical components mediating gene regulatory networks under Pi stress. In the present study, thirty-one transcription factor genes belonging to 10 diverse families including MYB/MYB-like, bHLH, WRKY and ERF were found to be responsive to Pi deprivation (Table 5). Many of these transcription factor families were previously found to be responsive to Pi starvation and mediate transcriptional regulation of Pi-responsive genes in plants [21,71,72,73,74]. The identification of these short-term Pi stress-responsive transcription factors may provide preliminary evidence for further characterization of their functions in early Pi stress signaling.

Many genes possibly involved in protein phosphorylation and dephosphorylation are transcriptionally affected by short-term Pi stress (Figure 4). This result conforms with previous reports in Arabidopsis, demonstrating that many kinds of kinase and phosphatase genes are differentially expressed upon Pi deficiency [21,22,75]. Protein phosphorylation and dephosphorylation are linked with phosphate transporter trafficking and metabolic acclimations under Pi deficiency stress [76,77]. Recently, rice kinases CK2 and PSTOL1 were illustrated to be involved in regulating Pi stress response and tolerance [78,79]. Arabidopsis plasma membrane-localized receptor-like kinase BIK1 (botrytis-induced kinase 1) and MKK9-MPK3/MPK6 (mitogen-activated protein kinase kinase9-mitogen-activated protein kinase3/mitogen-activated protein kinase6) cascade were also shown to have functions in Pi signaling [80,81]. Moreover, some kinases like CIPKs can regulate the activities of transporters of nutrients like nitrate and potassium [82,83]. It would be interesting to examine the roles of Pi-responsive kinases or phosphatases in the early Pi stress signaling.

The expressions of many genes affected by short-term Pi deprivation are associated with metabolisms, such as lipid metabolism, cell wall degradation and modification, carbohydrate metabolism and amino acid metabolism (Figure 5). Earlier studies also demonstrated these primary or secondary metabolic changes upon medium-term or long-term Pi deficiency in plants [21,22,27]. Thus the modifications in expression levels of genes involved in metabolism can exist from the early stage of Pi stress. Cell walls have crucial functions in regulating the rate and direction of growth and determining the morphology of plant cells and organs. Synthesis and remodeling of the cell wall were documented to be associated with the acclimations to many kinds of environmental stresses [84].

In conclusion, our RNA-seq analysis revealed an early transcriptomic response of soybean leaves to Pi deprivation, suggesting an intricate regulatory network of signaling components upon short-term Pi stress. Quick changes in the transcript levels of various genes allow the plant to properly and accurately acclimate to Pi limitation conditions. Although the exact roles of these early Pi stress-responsive genes remain to be investigated, our data provide a platform for further functional characterizations of these genes in Pi stress sensing, signaling and tolerance.

## 4. Materials and Methods

### 4.1. Plant Material and Growth Conditions

Williams 82 is the soybean cultivar used for the reference soybean genome [53]. Soybean seeds (*Glycine max* var. Williams 82) (kindly provided by Prof. Haijian Zhi from Nanjing Agricultural University) were soaked in sterilized water for 4 h and then incubated at room temperature in the dark between two layers of moistened filter paper. Four days later, seedlings were transferred and grown hydroponically in a 10 L tank filled with a half-strength modified Hoagland nutrient solution containing 2.5 mm Ca(NO_3_)_2_, 2.5 mm KNO_3_, 0.5 mm KH_2_PO_4_, 1.25 mm MgSO_4_, 10.0 μm Fe-EDTA, 3.4 μm MnSO_4_, 0.16 μm CuSO_4_, 0.38 μm ZnSO_4_, 23.0 μm H_3_BO_3_, 0.25 μm Na_2_MoO_4_, with pH adjusted to 5.6. Nutrient solution was changed every two days. Plants were grown in a growth chamber with a photoperiod set at 16-h-light/8-h-dark at 26/22 °C and light intensity set at 150 μL·m^−2^·s^−1^. After 18 days of cultivation in hydroponics, soybean seedlings with the first trifoliate true leaves fully expanded were transferred into Pi-sufficient (0.5 mm KH_2_PO_4_) or Pi-deprived (0 mm KH_2_PO_4_, K_2_SO_4_ was substituted for KH_2_PO_4_ to keep the concentration of K^+^) nutrient solutions. Roots and leaves of soybean seedlings were separately sampled after 24 h Pi deprivation, frozen in liquid nitrogen and stored at −80 °C until RNA preparation.

### 4.2. Pi Concentration Determination

Pi concentrations were analyzed as described previously [35]. About 0.2 g fresh tissue that were frozen in liquid nitrogen were ground into fine powder and suspended in extraction buffer (10 mm Tris, 1 mm EDTA, 100 mm NaCl and 1 mm β-mercaptoethanol, pH 8.0) at a ratio of 1 mg of fresh weight sample to 10 μL of extraction buffer. A total of 100 μL of sample suspension was mixed with 900 μL of 1% glacial acetic acid and incubated at 42 °C for 30 min. Then, the suspension was centrifuged at 13,000× *g* for 10 min and 500 μL of the supernatant was used for the Pi quantitation assay. The reaction mixture containing 1000 μL of Pi assay solution (0.34% (NH_4_)_6_Mo_7_O_24_·4H_2_O, 0.46 M H_2_SO_4_ and 1.4% ascorbic acid) and 500 μL of supernatant was incubated at 42 °C for 30 min, cooled on ice and the absorbance at 820 nm was measured using a UV-Vis spectrum meter (Thermo Scientific BioMate 3S, Chino, CA, USA).

### 4.3. RNA Isolation, Library Construction and RNA Sequencing

Soybean leaves (the first trifoliate true leaves) were collected after Pi-deprivation treatment for 24 h. Four samples (two biological replicates of both Pi-deprived and Pi-sufficient leaves) were used for mRNA library construction and sequencing. Each biological replicate was sampled from three different randomly-selected plants. Total RNA was extracted using Trizol reagent (Invitrogen, Carlsbad, CA, USA) following the manufacturer’s procedure. The total RNA quantity and purity were determined by Agilent Bioanalyzer 2100 with RNA 6000 Nano LabChip Kit (Agilent, Santa Clara, CA, USA). The RIN (RNA integrity number) of all RNA samples were determined to be more than 7.0. Approximately 10 µg of total RNA was subjected to Poly (A) mRNA isolation using poly-T oligo-attached magnetic beads (Invitrogen). The mRNA was fragmented into small pieces using divalent cations under elevated temperature after purification. The cleaved RNA fragments were reverse-transcribed to create the final cDNA library in accordance with the protocol for the mRNA-Seq sample preparation kit (Illumina, San Diego, CA, USA). The average insert size for the paired-end libraries was 300 ± 50 bp. Subsequently, paired-end sequencing was performed on an Illumina Hiseq 2000 at the LC-BIO TECHNOLOGIES (Hangzhou, China) following the instructions from Illumina.

### 4.4. RNA-Seq Reads Mapping and Differential Counting

The initial base calling and quality filtering of the reads generated with the Illumina analysis pipeline (Fastq format) were implemented using a custom Perl script and the default parameters of the Illumina pipeline (http://www.illumina.com). Additional filtering for poor-quality bases was carried out using the FASTX-toolkit available in the FastQC software package (http://www.bioinformatics.babraham.ac.uk/projects/fastqc/). To facilitate the read mapping, the *Glycine max* reference genome (Gmax2.0 version) was indexed by Bowtie2 (http://www.phytozome.net) [85]. The read mapping was conducted using the TopHat software package [86]. TopHat allows multiple alignments per read (up to 40) and a maximum of two mismatches when mapping the reads to the reference genome. The reads were first mapped directly to the genome using indexing and then the unmapped reads were used to identify novel splicing events. The aligned read files were processed by Cufflinks to measure the relative abundances of the transcripts by using the normalized RNA-seq fragment counts [87]. The estimated abundance of genes was measured in terms of the fragments per kilobase of transcript per million mapped reads (FPKM). Differentially expressed genes (DEGs) between the two sets of samples were identified using Cuffdiff [87]. Only the genes with a log2 fold change ≥1 or ≤−1 and a *p*-value ≤ 0.05 were considered as significantly DEGs.

The datasets were deposited in NCBI’s Gene Expression Omnibus and are accessible through GEO accession number GSE104286 (https://www.ncbi.nlm.nih.gov/geo/query/acc.cgi?acc=GSE104286) (the secure token for review is mbuhcgoylbanjyx).

### 4.5. Quantitative RT-PCR Analysis

Total RNA was extracted from soybean tissues using RNApure Plant Kit (with DNase I) (CoWin Biotech, Beijing, China) and digested with DNase I to eliminate genomic DNA contamination according to the manufacturer’s instruction. cDNA was synthesized from 1.0 μg total RNA in a 20 μL reaction by SuperRT Reverse Transcriptase (CoWin Biotech) using oligo (dT) primers. Quantitative RT-PCR (qRT-PCR) was performed on a MyiQ Single Color Real-time PCR system (Bio-Rad, Hercules, CA, USA) as described previously [88]. Briefly, two μL of a 1/10 dilution of cDNA in water was added to 10 μL of 2 × UltraSYBR (with Rox) (CoWin Biotech); together with two gene-specific primers (200 nm each) the final volume was brought to 20 uL by adding DNase-free water. The procedures for PCR were as follows:15 °C for 10 min; 40 cycles of 95 °C for 15 s, 60 °C for 60 s. Amplifications were run in triplicate together with controls that contained no template and no reverse transcription for each of the examined genes. Relative expression levels were normalized to that of an internal control *ACTIN11* (*Glyma.18g290800*) using the Pfaffl method (Ratio = (*E*_target_)^∆*C*t^_target_^(control−sample)^/(*E*_ref_)^∆*C*t^_ref_^(control−sample)^) [89]. The calculated efficiency (*E*) of all primers was between 1.7 and 2.2. The gene-specific primers are listed in Table S16.

### 4.6. Functional Annotation and Gene Ontology (GO) Enrichment

The DEGs were annotated for gene ontology (GO) terms [90] and categorized into molecular function, cellular component and biological process categories. A gene enrichment test was performed on each of the gene lists to acquire the terms that were significantly enriched among the DEGs. Fisher’s exact test, which is based on hyper-geometric distribution, was used to calculate the *p*-value. A GO category (http://geneontology.org/) or KEGG pathway (http://www.genome.jp/kegg/) with a *p*-value ≤ 0.05 was regarded as significantly enriched. GO and KEGG enrichment analyses were conducted with the help of LC-BIO company (Hangzhou, China).

The MultiExperiment Viewer program (http://mev.tm4.org/) was used to draw a heatmap of the significant DEGs in response to short-term Pi deprivation. The metabolic pathways were plotted using MapMan (http://mapman.gabipd.org/) [91].

### 4.7. Promoter Cis-Element Analysis

Promoter sequences (1000 bp upstream) of the transcription start sites of DEGs were extracted from the SoyBase database (http://www.soybase.org/). The presence of the Pi-responsive cis-elements was analyzed using Regulatory Sequence Analysis Tools (http://floresta.eead.csic.es/rsat/) [92]. The sequences of eight types of Pi-responsive cis-elements were as follows: P1BS (GNATATNC) [11]; PHO (CACGT(G/C)); PHO-like (C(G/T/A)(C/T/A)GTGG) [19]; P responsive (ATGCCAT) [43]; TATA box like (TATAAATA) [19]; TC element (TCTCTCT); NIT 2-like (AAATATCT); Helix-loop-helix (CA(T/G)(A/C)TG) [41].

## Figures and Tables

**Table 1:** Differentially expressed genes where transcripts were found only in Pi-deprived leaves (PDL) or Pi-sufficient leaves (PSL) after 24 h Pi deprivation. Expression level of each gene was measured in terms of fragments per kilobase of transcript per million mapped reads (FPKM). Gene functional descriptions were shown according to soybean genome annotation (V2.0). Accession number “Glyma.” is named as “Gm” for short. −P refers to PDL and +P refers to PSL. NA: no annotation.

Accession No.	−P (FPKM)	+P (FPKM)	*p* Value	Description
Gm04g223400	1260.24	0.00	1.98 × 10^−2^	NA
Gm17g100000	21.71	0.00	1.65 × 10^−3^	mini zinc finger
Gm17g090400	3.82	0.00	3.95× 10^−3^	calcium-dependent protein kinase 6
Gm16g028300	2.72	0.00	4.10 × 10^−2^	NA
Gm15g099200	2.27	0.00	2.00 × 10^−4^	NA
Gm06g240900	1.25	0.00	1.50 × 10^−2^	PLATZ transcription factor family protein
Gm09g023000	1.11	0.00	5.00 × 10^−5^	peroxidase superfamily protein
Gm06g177200	0.77	0.00	2.40 × 10^−3^	NA
Gm19g246600	0.75	0.00	1.00 × 10^−4^	SSXT family protein
Gm13g068300	0.00	47.11	9.65 × 10^−3^	NA
Gm05g099700	0.00	10.71	3.85 × 10^−3^	NA
Gm04g128500	0.00	5.87	7.50 × 10^−4^	late embryogenesis abundant protein, group 1 protein
Gm13g236300	0.00	5.77	9.00 × 10^−4^	NA
Gm04g014000	0.00	3.30	5.00 × 10^−5^	polyol/monosaccharide transporter 5
Gm13g178000	0.00	2.18	4.19 × 10^−2^	NA
Gm07g022900	0.00	2.11	8.65 × 10^−3^	phospholipid *N*-methyltransferase
Gm04g094800	0.00	1.21	5.00 × 10^−5^	plant-specific transcription factor YABBY family protein
Gm11g179100	0.00	1.02	5.00 × 10^−5^	aluminum activated malate transporter family protein
Gm03g113600	0.00	0.82	5.00 × 10^−5^	auxin efflux carrier family protein
Gm04g044300	0.00	0.80	1.61 × 10^−2^	cytochrome B5 isoform D
Gm15g197300	0.00	0.73	5.00 × 10^−5^	CBL-interacting protein kinase 23

**Table 2:** DEGs potentially involved in Pi signaling and utilization. Expression level of each gene was measured in terms of FPKM. Gene functional descriptions were shown according to soybean genome annotation (V2.0). Accession number “Glyma.” is named as “Gm” for short. −P refers to PDL and +P refers to PSL. SPX: domain found in Syg1, Pho81, XPR1, and related proteins; HAD: haloacid dehydrogenase.

Accession No.	−P (FPKM)	+P (FPKM)	Log2(−P/+P)	*p* Value	Description
Gm03g078300	1.59	0.09	4.15	4.42 × 10^−2^	Sulfoquinovosyldiacylglycerol 2 (*GmSQD2*)
Gm04g147600	2.80	0.41	2.76	1.03 × 10^−2^	SPX domain-containing protein 1-related (*GmSPX3*)
Gm08g056400	9.38	1.43	2.71	5.00 × 10^−5^	Purple acid phosphatase (*GmPAP13*)
Gm01g091800	0.90	0.22	2.02	4.90 × 10^−3^	Phosphate transporter PHO1 homolog 1 (*GmPHO1*;*H12*)
Gm02g130200	1.28	0.32	2.02	6.50 × 10^−4^	Phosphate transporter PHO1 homolog 1 (*GmPHO1*;*H14*)
Gm20g238000	1.42	0.38	1.89	1.00 × 10^−4^	Phospholipase D P1 (*GmPLDZ2*)
Gm06g069000	2.68	0.91	1.56	2.52 × 10^−2^	SPX domain-containing protein 1-related (*GmSPX4*)
Gm17g114700	19.96	7.53	1.41	5.00 × 10^−5^	SPX domain-containing protein 1-related (*GmSPX8*)
Gm08g194100	0.71	0.29	1.29	2.20 × 10^−2^	Phospholipase D α 4
Gm01g002400	13.80	6.68	1.05	1.31 × 10^−2^	Phospholipase A2 family protein
Gm09g223700	34.43	16.85	1.03	5.00 × 10^−5^	Glycerol-3-phosphate permease gene family
Gm19g026600	2.78	1.16	1.27	1.40 × 10^−3^	Purple acid phosphatase (*GmPAP31*)
Gm16g220900	2.14	4.75	−1.15	1.64 × 10^−2^	HAD superfamily, subfamily IIIB acid phosphatase

**Table 3:** DEGs potentially involved in the transportation of water, sugars and mineral nutrients. Expression level of each gene was measured in terms of FPKM. Gene functional descriptions were shown according to soybean genome annotation (V2.0). Accession number “Glyma.” is named as “Gm” for short. −P refers to PDL and +P refers to PSL. ATOX1: antioxidant protein 1; PIP1: plasma membrane intrinsic protein 1; TIP2: tonoplast intrinsic aquaporin 2; ATP: adenosine triphospate; ABC: ATP-binding cassette.

Substrate	Accession No.	−P (FPKM)	+P (FPKM)	Log2(−P/+P)	*p* Value	Description
Sugar	Gm15g105900	1.48	0.47	1.64	2.78 × 10^−^^2^	Glucose-6-phosphate/phosphate translocator 2
	Gm13g206700	6.26	2.77	1.18	9.00 × 10^−^^4^	Glucose-6-phosphate/phosphate translocator 2
	Gm11g226200	131.29	63.50	1.05	5.00 × 10^−^^5^	Nucleotide-sugar transporter family protein
	Gm04g014000	0.00	3.30	+P only	5.00 × 10^−^^5^	Polyol/monosaccharide transporter 5
	Gm08g009900	2.80	19.84	−2.83	5.00 × 10^−^^5^	Bidirectional sugar transporter SWEET10
Zinc/iron	Gm16g168200	7.32	2.14	1.78	1.40 × 10^−^^3^	Vacuolar iron transporter (VIT) family protein
	Gm13g004400	1.33	7.82	−2.55	5.00 × 10^−^^5^	Zinc transporter 3-related protein
	Gm20g063100	1.80	9.06	−2.33	5.00 × 10^−^^5^	Zinc transporter 3-related protein
	Gm06g052000	24.56	58.72	−1.26	5.00 × 10^−^^5^	Zinc/iron transporter
Sulfate	Gm01g175200	2.16	0.83	1.37	3.55 × 10^−^^3^	Sulfite exporter TauE/SafE family protein
	Gm15g014000	1.78	0.84	1.08	1.60 × 10^−^^2^	Sulfate transporter 3;4
Copper	Gm08g180500	4.15	0.81	2.36	4.03 × 10^−^^2^	Copper transport protein ATOX1-related
	Gm14g072100	6.81	2.58	1.40	5.00 × 10^−^^5^	Copper transport protein ATOX1-related
	Gm15g051900	28.02	13.36	1.07	1.85 × 10^−^^3^	Copper transport protein ATOX1-related
Nitrate	Gm18g127200	1.07	3.08	−1.53	4.50 × 10^−^^4^	Nitrate transporter 1.7
Malate	Gm11g179100	0.00	1.02	+P only	5.00 × 10^−^^5^	Aluminum activated malate transporter
Water	Gm08g015300	8.72	3.58	1.29	3.50 × 10^−^^4^	Aquaporin PIP1;4-related
	Gm05g208700	15.22	6.50	1.23	1.50 × 10^−^^4^	Aquaporin PIP1;4-related
	Gm15g018100	69.65	33.30	1.06	5.00 × 10^−^^5^	Aquaporin TIP2;1
Unknown	Gm07g081000	18.37	4.44	2.05	5.00 × 10^−^^5^	ABC transporter family protein
	Gm11g106300	54.15	17.68	1.61	5.00 × 10^−^^5^	Major facilitator superfamily protein
	Gm04g027800	1.28	0.35	1.86	2.71 × 10^−^^2^	Protein Walls Are Thin 1
	Gm07g094000	0.43	2.97	−2.77	5.30 × 10^−^^3^	ATP-binding cassette A2
	Gm11g064100	0.87	2.05	−1.23	2.15 × 10^−^^3^	Major facilitator superfamily protein
	Gm08g101500	1.01	2.36	−1.22	5.00 × 10^−^^5^	Multidrug resistance-associated protein 3
	Gm17g165200	5.68	11.51	−1.02	5.00 × 10^−^^5^	Metal transporter NRAMP2-related
	Gm10g276700	2.39	4.79	−1.00	6.00 × 10^−^^4^	Major facilitator superfamily protein

**Table 4:** DEGs possibly related to Ca^2+^ and hormonal signaling. Expression level of each gene was measured in terms of FPKM. Gene functional descriptions were shown according to soybean genome annotation (V2.0). Accession number “Glyma.” is named as “Gm” for short. −P refers to PDL and +P refers to PSL. VCX1: vacuolar Ca^2+^ exchanger 1; AUX: auxin; IAA: indole-3-acetic acid; GA: gibberellin; BES1/BZR1: brassinosteroid-insensitive1-ethyl methanesulfonate-suppressor 1/brassinazole-resistant 1.

Ca^2+^/Hormone	Accession No.	−P (FPKM)	+P (FPKM)	Log2(−P/+P)	*p* Value	Description
Calcium	Gm02g226500	1.10	0.24	2.19	2.14 × 10^−^^2^	Ca^2+^/H^+^ antiporter VCX1 and related protein
	Gm14g000100	1.59	6.01	−1.92	5.00 × 10^−^^5^	Glutamate receptor 3.1-related protein
	Gm16g061800	0.37	0.83	−1.15	1.52 × 10^−^^2^	Glutamate receptor 2.5
	Gm07g203700	1.58	3.28	−1.06	2.50 × 10^−^^4^	Glutamate receptor 2.7
	Gm17g090400	3.82	0.00	−P only	3.95 × 10^−^^3^	Calcium-dependent protein kinase 6
	Gm17g130900	4.20	1.38	1.60	5.00 × 10^−^^4^	Calmodulin-binding protein IQ-domain 22
	Gm09g225300	1.10	0.37	1.57	1.30 × 10^−^^2^	Protein IQ-domain 11
	Gm01g002400	13.80	6.68	1.05	1.31 × 10^−^^2^	Phospholipase A2 family protein
	Gm18g230800	1.07	2.74	−1.36	5.00 × 10^−^^5^	Ca^2+^/lipid-binding phosphoribosyltransferase
	Gm09g236800	8.07	16.28	−1.01	1.70 × 10^−^^3^	Ca^2+^-binding EF-hand family protein (GmCML116)
Auxin	Gm16g115500	2.69	1.15	1.23	1.65 × 10^−^^2^	Auxin efflux carrier family protein
	Gm03g113600	0.00	0.82	+P only	5.00 × 10^−^^5^	Auxin efflux carrier family protein
	Gm19g168500	0.50	1.59	−1.66	2.75 × 10^−^^2^	Auxin-responsive protein IAA10-related
	Gm19g161100	9.82	25.41	−1.37	5.00 × 10^−^^5^	AUX/IAA family
Cytokinin	Gm04g247800	3.32	1.03	1.70	1.80 × 10^−^^2^	Response regulator of two-component system
	Gm05g144500	0.46	1.09	−1.22	1.58 × 10^−^^2^	Response regulator of two-component system
Gibberellin	Gm06g044400	439.61	89.83	2.29	5.00 × 10^−^^5^	Gibberellin-regulated family protein
	Gm06g185300	71.42	15.60	2.19	1.00 × 10^−^^4^	Gibberellin-regulated family protein
	Gm05g034500	19.80	6.63	1.58	1.89 × 10^−^^2^	Gibberellin-regulated family protein
	Gm06g193800	174.53	71.57	1.29	2.00 × 10^−^^4^	Gibberellin-regulated family protein
	Gm17g092800	17.65	7.92	1.16	4.94 × 10^−^^2^	Gibberellin-regulated family protein
	Gm15g002200	5.27	2.62	1.01	1.35 × 10^−^^3^	GA requiring 3
Brassinosteroid	Gm14g127400	1.36	0.24	2.52	1.28 × 10^−^^2^	BES1/BZR1 homolog protein 1
Jasmonate	Gm01g204400	4.22	1.71	1.30	4.99 × 10^−^^2^	JASMONATE-ZIM-domain protein 2-related
Ethylene	Gm10g008500	2.20	4.84	−1.14	2.61 × 10^−^^2^	Ethylene response sensor 2-related

**Table 5:** Transcription factor genes differentially expressed in soybean leaves under short-term Pi deprivation. Expression level of each gene was measured in terms of FPKM. Gene functional descriptions were shown according to soybean genome annotation (V2.0). Accession number “Glyma.” is named as “Gm” for short. −P refers to PDL and +P refers to PSL. MYB: (myeloblastosis); DOF: DNA-binding one zinc finger; YABBY: YABBY domain;CCT: CONSTANS, CO-like, and TOC1; NAC: NAM, ATAF, and CUC; TCP: a family of transcription factors named after: teosinte branched 1 (tb1, Zea mays (Maize)), cycloidea (cyc) (Antirrhinum majus) (Garden snapdragon) and PCF in rice (Oryza sativa); ERF: ethylene response factor; WRKY: a protein domain composed of a conserved WRKYGQK motif; RING: really interesting new gene.

Accession No.	−P (FPKM)	+P (FPKM)	Log2(−P/+P)	*p* Value	Description
Gm14g127400	1.36	0.24	2.52	1.28 × 10^−2^	BES1/BZR1 homolog protein 1-related
Gm17g257700	1.25	0.31	2.00	4.86 × 10^−2^	C2H2-like zinc finger protein
Gm08g215400	1.86	0.47	1.99	3.36 × 10^−2^	Basic helix-loop-helix (bHLH) DNA-binding superfamily protein
Gm06g284900	1.93	0.54	1.82	3.15 × 10^−3^	Basic-leucine zipper (bZIP) transcription factor family protein
Gm16g017700	4.93	1.56	1.66	2.50 × 10^−4^	Basic helix-loop-helix (bHLH) DNA-binding superfamily protein
Gm20g186500	12.12	4.34	1.48	5.00 × 10^−5^	MYB-like transcription factor family protein
Gm12g037200	7.12	2.78	1.36	1.59 × 10^−2^	B-box type zinc finger family protein
Gm07g012100	8.87	3.59	1.30	2.10 × 10^−3^	DOF zinc finger protein 1
Gm18g042300	2.28	0.94	1.29	5.35 × 10^−3^	Zinc finger protein 4
Gm13g061900	1.19	0.51	1.22	4.94 × 10^−2^	MYB domain protein 16
Gm05g056000	5.89	2.62	1.17	1.16 × 10^−2^	Plant-specific transcription factor YABBY family protein
Gm01g133500	7.16	3.43	1.06	4.33 × 10^−2^	Basic helix-loop-helix (bHLH) DNA-binding superfamily protein
Gm11g117100	6.84	3.35	1.03	1.05 × 10^−3^	Basic helix-loop-helix (bHLH) DNA-binding superfamily protein
Gm04g094800	0.00	1.21	+P only	5.00 × 10^−5^	Plant-specific transcription factor YABBY family protein
Gm13g203700	0.13	1.03	−3.02	4.61 × 10^−2^	C2H2-type zinc finger family protein
Gm01g029300	1.37	6.15	−2.16	3.77 × 10^−2^	Plant-specific transcription factor YABBY family protein
Gm10g021400	0.35	1.44	−2.06	2.51 × 10^−2^	B-box type zinc finger protein with CCT domain
Gm11g182000	0.92	3.34	−1.87	1.33 × 10^−2^	NAC domain containing protein 90
Gm13g047400	0.31	1.10	−1.85	1.38 × 10^−2^	TCP family transcription factor
Gm12g162700	0.62	2.11	−1.76	2.43 × 10^−2^	ERF domain protein 9
Gm02g177800	0.85	2.71	−1.67	3.78 × 10^−2^	MYB-like HTH transcriptional regulator family protein
Gm05g002700	0.29	0.89	−1.62	4.98 × 10^−2^	NAC (No Apical Meristem) domain transcriptional regulator
Gm01g041700	1.09	2.98	−1.46	7.15 × 10^−3^	Homeobox 1
Gm02g217800	4.27	11.31	−1.40	1.00 × 10^−4^	Transcription factor bHLH61-related
Gm03g221700	1.40	3.66	−1.39	2.28 × 10^−2^	MYB domain protein 2
Gm08g256400	1.43	3.54	−1.31	7.20 × 10^−3^	TCP family transcription factor
Gm02g152900	0.94	2.25	−1.25	2.90 × 10^−2^	B-box type zinc finger protein with CCT domain
Gm05g144500	0.46	1.09	−1.22	1.58 × 10^−2^	Response regulator 11
Gm07g227200	6.34	12.74	−1.01	1.50 × 10^−4^	WRKY DNA-binding protein 3
Gm08g286000	1.41	2.83	−1.00	1.25 × 10^−2^	Zinc finger (CCCH-type/C3HC4-type RING finger) family protein

## Supplementary Materials

Supplementary materials can be found at http://www.mdpi.com/1422-0067/19/7/2145/s1.
