# Supplementary material for: Early Transcriptomic Response to Phosphate Deprivation in Soybean Leaves as Revealed by RNA-Sequencing
Source: Int J Mol Sci. 2018 Jul 23;19(7):2145. doi: 10.3390/ijms19072145 (PMC6073939; doi:10.3390/ijms19072145)
Supplement: Supplementary file 1 [file ijms-19-02145-s001.zip › Supplemental figures.pdf]

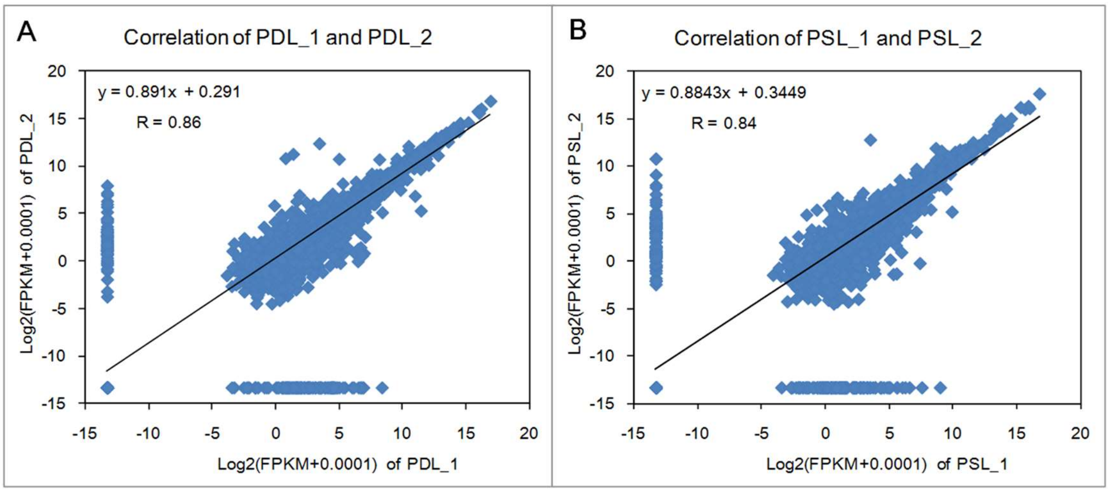

Figure S1. Pearson's correlation (R value) of two biological replicates. A, two biological replicates of Pi-deprived leaves (PDL_1 and PDL_2). B, two biological replicates of Pi-sufficient leaves (PSL_1 and PSL_2).

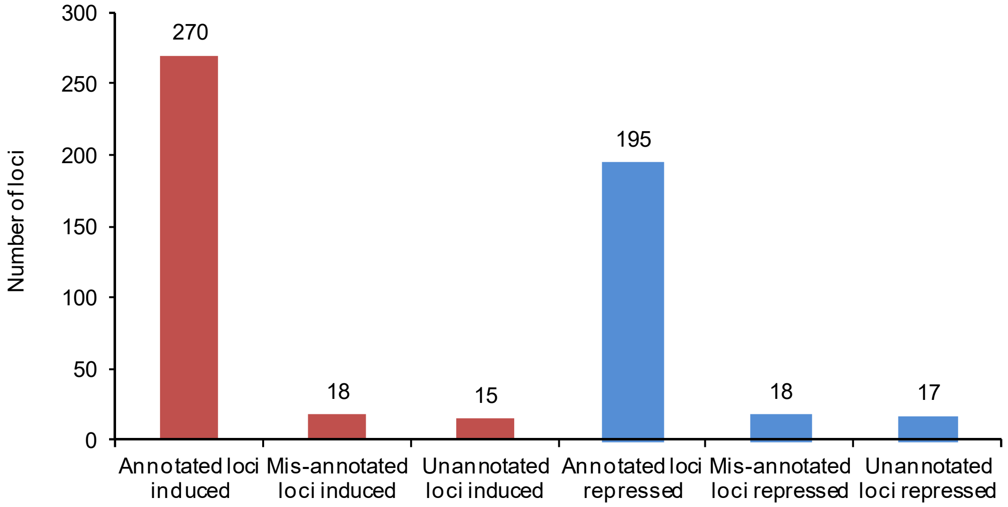

Figure S2. Histogram representing the differentially expressed genes between Pi-deprived and Pi-sufficient leaves. Red and blue colors indicate up- and down-regulated genes, respectively, under Pi deprivation.

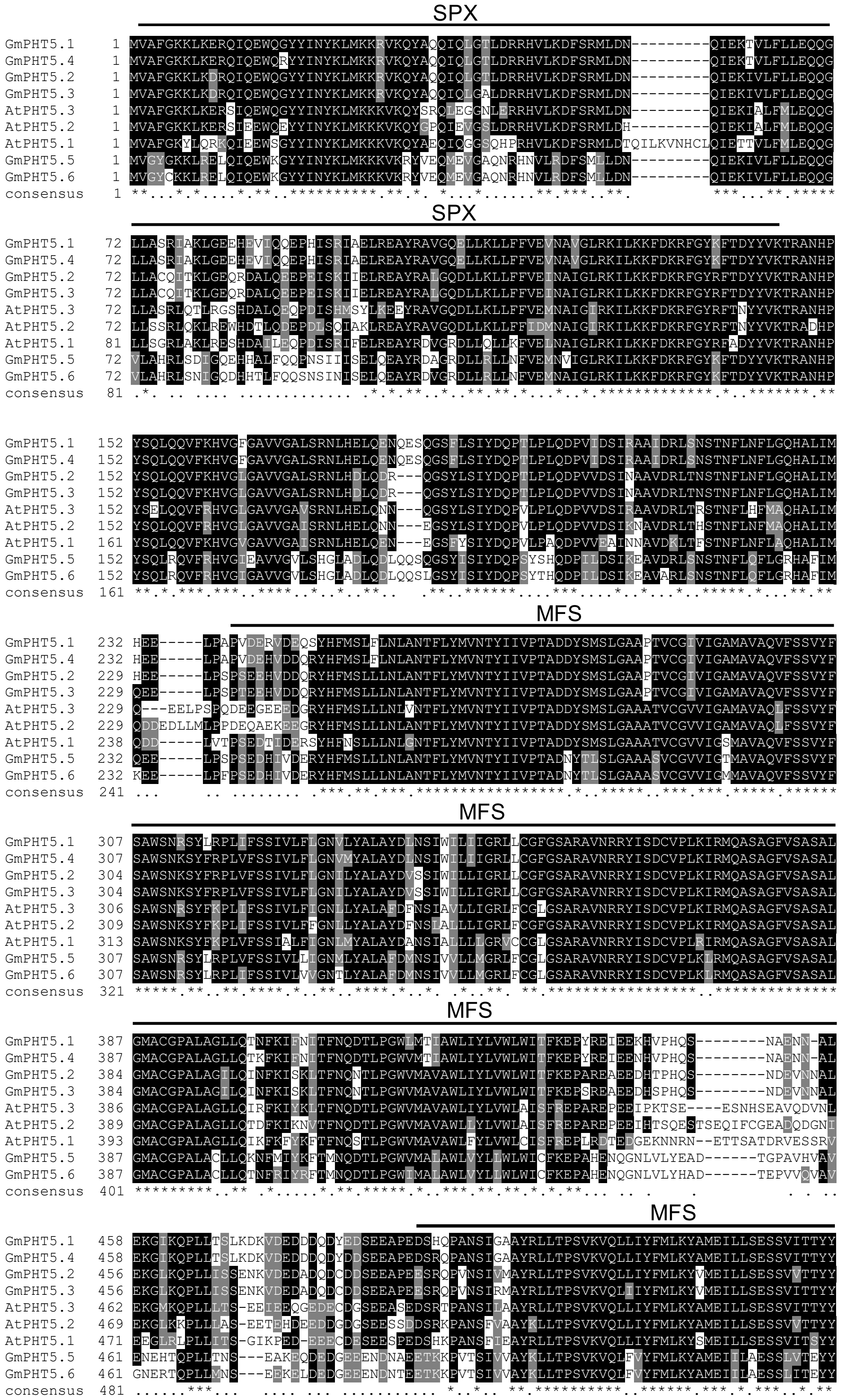
SPX
SPX
MFS
MFS
MFS
MFS

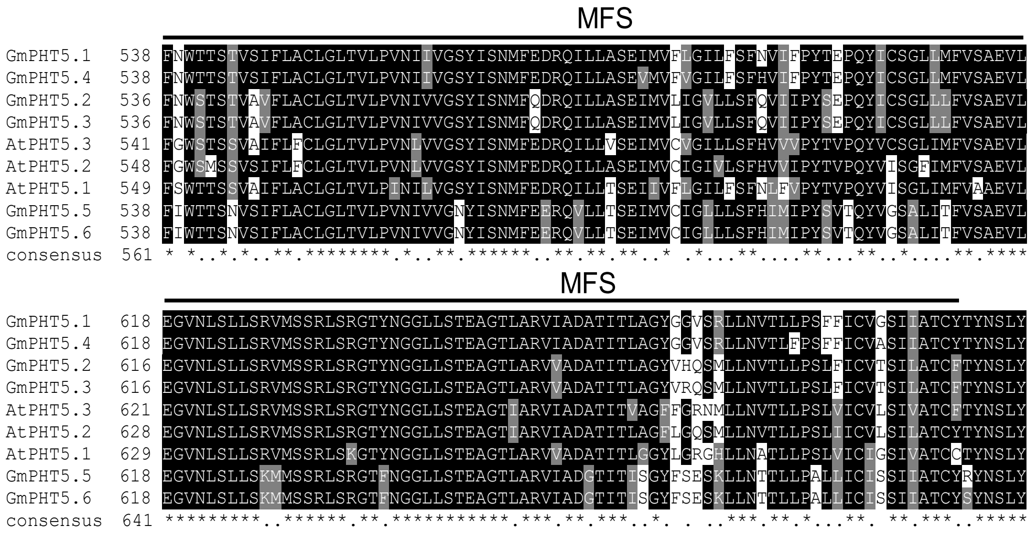

Figure S3. Alignment of the amino acid sequences of the six soybean SPX-MFS proteins and the three Arabidopsis SPX-MFS proteins with ClustalW (http://www.genome.jp/tools/clustalw/) and BoxShade (http://www.ch.embnet.org/software/BOX_form.html). Black blocks indicate highly conserved residues. Dashes indicate gaps introduced to allow for optimal alignment of the sequences. The SPX domain and the MFS domain are indicated by lines.

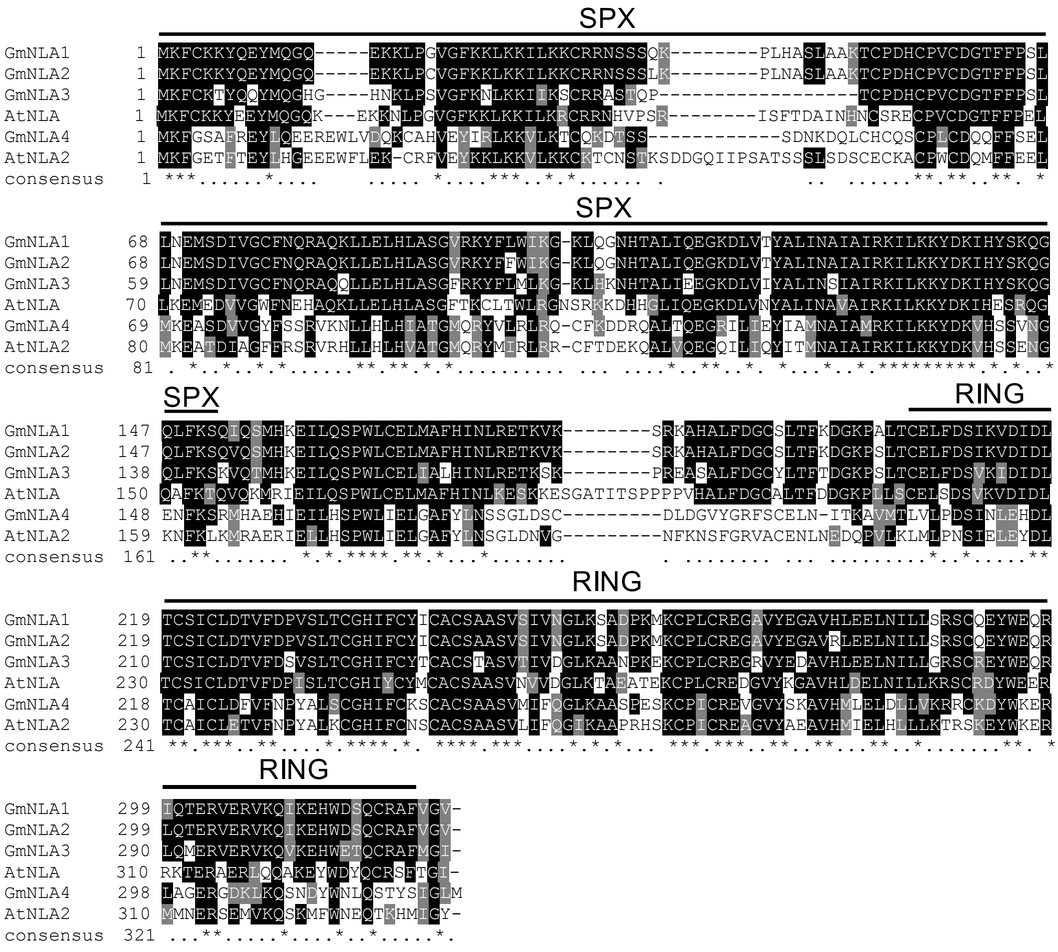

Figure S4. Alignment of the amino acid sequences of the four soybean SPX-RING proteins and the two Arabidopsis SPX-RING proteins with ClustalW (http://www.genome.jp/tools/clustalw/) and BoxShade (http://www.ch.embnet.org/software/BOX_form.html). Black blocks indicate highly conserved residues. Dashes indicate gaps introduced to allow for optimal alignment of the sequences. The SPX domain and the RING domain are indicated by lines.

A

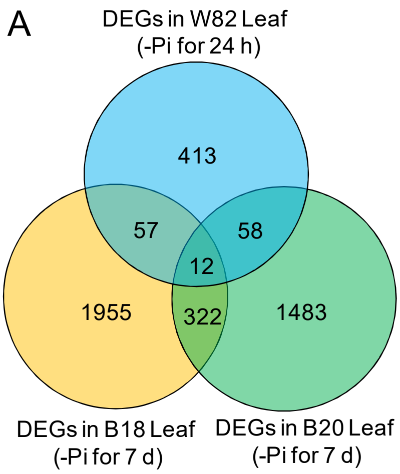

B

Gene	-P 7d in B18 leaf log2(-P/+P)	-P 7d in B20 leaf log2(-P/+P)	-P 24h in W82 leaf log2(-P/+P)	Description
Glyma.04G019500	1.13	1.32	1.05	SKU5 similar 5
Glyma.05G076700	1.17	1.07	1.18	Cupredoxin superfamily protein
Glyma.08G109500	-2.90	1.29	-1.06	Chalcone and stilbene synthase family protein
Glyma.08G195100	-3.69	5.14	2.24	Pyridoxal phosphate phosphatase-related protein
Glyma.12G229000	-1.14	1.49	-1.17	cytochrome BC1 synthesis
Glyma.13G206700	-1.17	1.36	1.18	glucose-6-phosphate/phosphate translocator 2
Glyma.14G175800	1.20	1.61	1.22	UDP-glucosyl transferase 85A3
Glyma.17G214500	-1.36	1.19	-1.41	Protein kinase superfamily protein
Glyma.18G065100	2.26	2.06	1.69	Laccase/Diphenol oxidase family protein
Glyma.18G127200	-2.83	-1.15	-1.53	nitrate transporter 1.7
Glyma.19G161100	-1.02	1.08	-1.37	indole-3-acetic acid inducible 14
Glyma.19G223000	1.67	-5.92	1.21	Glycosyl hydrolase superfamily protein

Figure S5. Genes that are common responsive to short-term Pi deprivation (24 h) and long-term Pi deprivation (7 d) in soybean leaves of different genotypes. B20 is a low-P tolerant genotype, while B18 is a low-P sensitive genotype (Zhang et al 2017 Plant Mol Biol 93:137-150). Soybean cultivar Williams 82 (W82) was used in this study. (A) Venn diagram illustrating 12 common differentially expressed genes (DEGs) in the three genotypes. (B) List of the 12 common DEGs in the three genotypes under short-term or long-term Pi deprivation.

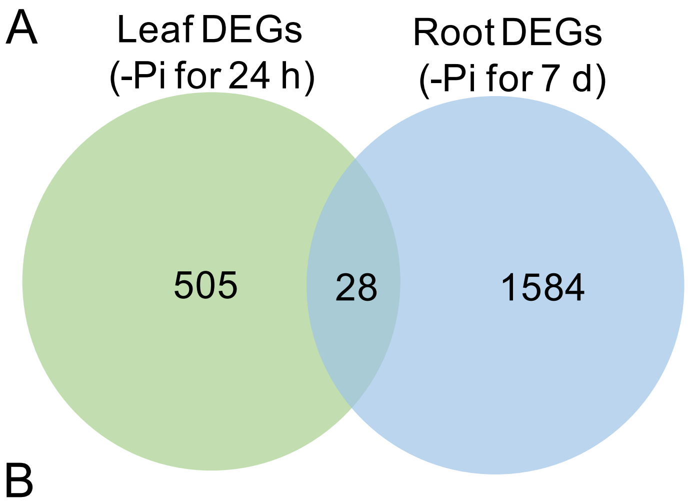

B

Gene	-P 24 h in leaf Log2(-P/+P)	-P 7 d in root Log2(-P/+P)	Description
Glyma.17G122900	8.65	-2.52	NA
Glyma.19G098500	3.85	1.70	Senescence-related gene 3
Glyma.11G046700	2.79	-1.98	NA
Glyma.04G147600	2.76	4.63	SPX domain gene 2
Glyma.12G065500	2.63	3.67	NA
Glyma.13G113000	2.32	-P only	NA
Glyma.20G238000	1.89	2.26	Phospholipase D P1
Glyma.08G203700	1.81	-3.48	Isochorismatase family protein
Glyma.04G247800	1.70	-4.84	Response regulator 9
Glyma.12G037300	1.36	-3.45	NA
Glyma.10G194000	1.34	-6.85	UDP-Glycosyltransferase superfamily protein
Glyma.08G154800	1.24	2.72	Putative methyltransferase family protein
Glyma.19G145700	1.08	-P only	Pectin lyase-like superfamily protein
Glyma.09G223700	1.03	2.05	Phosphate starvation-induced gene 3
Glyma.17G159100	-7.43	-2.88	NA
Glyma.19G148400	-3.67	-P only	NA
Glyma.07G094000	-2.77	5.04	ATP-binding cassette A2
Glyma.13G004400	-2.55	-1.11	Zinc transporter 5 precursor
Glyma.05G176900	-1.91	-P only	NA
Glyma.16G131200	-1.58	-3.16	Ferulic acid 5-hydroxylase 1
Glyma.11G074100	-1.37	-2.03	Ferulic acid 5-hydroxylase 1
Glyma.13G232800	-1.30	-4.12	RING/U-box superfamily protein
Glyma.17G072200	-1.09	-1.12	Cellulose synthase family protein
Glyma.03G145600	-1.06	-1.32	Peroxidase 2
Glyma.16G178800	-1.05	2.57	Heat shock protein 90.1
Glyma.17G165200	-1.02	-2.37	Natural resistance-associated macrophage protein 3
Glyma.06G158800	-1.01	-1.16	Eukaryotic aspartyl protease family protein
Glyma.14G222700	-1.01	-1.78	Prolyl oligopeptidase family protein

Figure S6. Soybean genes that are common responsive to short-term Pi deprivation (24 h) in leaves in this study and long-term Pi deprivation (7 d) in roots in previous study (Zeng et al 2016 Plant and soil 398: 207-27). (**A**) Venn diagram illustrating 28 common differentially expressed genes (DEGs) in leaves and roots. (**B**) List of the common DEGs.
